# Erectile Dysfunction Severity as a Risk Marker for Cardiovascular Disease Hospitalisation and All-Cause Mortality: A Prospective Cohort Study

**DOI:** 10.1371/journal.pmed.1001372

**Published:** 2013-01-29

**Authors:** Emily Banks, Grace Joshy, Walter P. Abhayaratna, Leonard Kritharides, Peter S. Macdonald, Rosemary J. Korda, John P. Chalmers

**Affiliations:** 1 National Centre for Epidemiology and Population Health, Australian National University, Canberra, Australian Capital Territory, Australia; 2 The Sax Institute, Sydney, New South Wales, Australia; 3 College of Medicine, Biology and the Environment, Australian National University, Canberra, Australian Capital Territory, Australia; 4 Concord Repatriation General Hospital, Sydney Medical School, University of Sydney, Sydney, New South Wales, Australia; 5 Victor Chang Cardiac Research Institute, Sydney, New South Wales, Australia; 6 The George Institute for Global Health, University of Sydney, Sydney, New South Wales, Australia; London School of Hygiene & Tropical Medicine, United Kingdom

## Abstract

**Background:**

Erectile dysfunction is an emerging risk marker for future cardiovascular disease (CVD) events; however, evidence on dose response and specific CVD outcomes is limited. This study investigates the relationship between severity of erectile dysfunction and specific CVD outcomes.

**Methods and Findings:**

We conducted a prospective population-based Australian study (the 45 and Up Study) linking questionnaire data from 2006–2009 with hospitalisation and death data to 30 June and 31 Dec 2010 respectively for 95,038 men aged ≥45 y. Cox proportional hazards models were used to examine the relationship of reported severity of erectile dysfunction to all-cause mortality and first CVD-related hospitalisation since baseline in men with and without previous CVD, adjusting for age, smoking, alcohol consumption, marital status, income, education, physical activity, body mass index, diabetes, and hypertension and/or hypercholesterolaemia treatment. There were 7,855 incident admissions for CVD and 2,304 deaths during follow-up (mean time from recruitment, 2.2 y for CVD admission and 2.8 y for mortality). Risks of CVD and death increased steadily with severity of erectile dysfunction. Among men without previous CVD, those with severe versus no erectile dysfunction had significantly increased risks of ischaemic heart disease (adjusted relative risk [RR] = 1.60, 95% CI 1.31–1.95), heart failure (8.00, 2.64–24.2), peripheral vascular disease (1.92, 1.12–3.29), “other” CVD (1.26, 1.05–1.51), all CVD combined (1.35, 1.19–1.53), and all-cause mortality (1.93, 1.52–2.44). For men with previous CVD, corresponding RRs (95% CI) were 1.70 (1.46–1.98), 4.40 (2.64–7.33), 2.46 (1.63–3.70), 1.40 (1.21–1.63), 1.64 (1.48–1.81), and 2.37 (1.87–3.01), respectively. Among men without previous CVD, RRs of more specific CVDs increased significantly with severe versus no erectile dysfunction, including acute myocardial infarction (1.66, 1.22–2.26), atrioventricular and left bundle branch block (6.62, 1.86–23.56), and (peripheral) atherosclerosis (2.47, 1.18–5.15), with no significant difference in risk for conditions such as primary hypertension (0.61, 0.16–2.35) and intracerebral haemorrhage (0.78, 0.20–2.97).

**Conclusions:**

These findings give support for CVD risk assessment in men with erectile dysfunction who have not already undergone assessment. The utility of erectile dysfunction as a clinical risk prediction tool requires specific testing.

*Please see later in the article for the Editors' Summary*

## Abstract 2

In a prospective Australian population-based study linking questionnaire data from 2006–2009 with hospitalisation and death data to June 2010 for 95,038 men aged ≥45 years, Banks and colleagues found that more severe erectile dysfunction was associated with higher risk of cardiovascular disease.

## Editors' Summary

**Background:**

Erectile dysfunction is the medical term used when a man is unable to achieve or sustain an erection of his penis suitable for sexual intercourse. Although a sensitive topic that can cause much embarrassment and distress, erectile dysfunction is very common, with an estimated 40% of men over the age of 40 years experiencing frequent or occasional difficulties. The most common causes of erectile dysfunction are medications, chronic illnesses such as diabetes, and drinking too much alcohol. Stress and mental health problems can also cause or worsen erectile dysfunction. There is also increasing evidence that erectile dysfunction may actually be a symptom of cardiovascular disease—a leading cause of death worldwide—as erectile dysfunction could indicate a problem with blood vessels or poor blood flow commonly associated with cardiovascular disease.

**Why Was This Study Done?:**

Although previous studies have suggested that erectile dysfunction can serve as a marker for cardiovascular disease in men not previously diagnosed with the condition, few studies to date have investigated whether erectile dysfunction could also indicate worsening disease in men already diagnosed with cardiovascular disease. In addition, previous studies have typically been small and have not graded the severity of erectile dysfunction or investigated the specific types of cardiovascular disease associated with erectile dysfunction. In this large study conducted in Australia, the researchers investigated the relationship of the severity of erectile dysfunction with a range of cardiovascular disease outcomes among men with and without a previous diagnosis of cardiovascular disease.

**What Did the Researchers Do and Find?:**

The researchers used information from the established 45 and Up Study, a large cohort study that includes 123,775 men aged 45 and over, selected at random from the general population of New South Wales, a large region of Australia. A total of 95,038 men were included in this analysis. The male participants completed a postal questionnaire that included a question on erectile functioning, which allowed the researchers to define erectile dysfunction as none, mild, moderate, or severe. Using information captured in the New South Wales Admitted Patient Data Collection—a complete record of all public and private hospital admissions, including the reasons for admission and the clinical diagnosis—and the government death register, the researchers were able to determine health outcomes of all study participants. They then used a statistical model to estimate hospital admissions for cardiovascular disease events for different levels of erectile dysfunction.

The researchers found that the rates of severe erectile dysfunction among study participants were 2.2% for men aged 45–54 years, 6.8% for men aged 55–64 years, 20.2% for men aged 65–74 years, 50.0% for men aged 75–84 years, and 75.4% for men aged 85 years and over. During the study period, the researchers recorded 7,855 hospital admissions related to cardiovascular disease and 2,304 deaths. The researchers found that among men without previous cardiovascular disease, those with severe erectile dysfunction were more likely to develop ischemic heart disease (risk 1.60), heart failure (risk 8.00), peripheral vascular disease (risk 1.92), and other causes of cardiovascular disease (risk 1.26) than men without erectile dysfunction. The risks of heart attacks and heart conduction problems were also increased (1.66 and 6.62, respectively). Furthermore, the combined risk of all cardiovascular disease outcomes was 1.35, and the overall risk of death was also higher (risk 1.93) in these men. The researchers found that these increased risks were similar in men with erectile dysfunction who had previously been diagnosed with cardiovascular disease.

**What Do These Findings Mean?:**

These findings suggest that compared to men without erectile dysfunction, there is an increasing risk of ischemic heart disease, peripheral vascular disease, and death from all causes in those with increasing degrees of severity of erectile dysfunction. The authors emphasize that erectile dysfunction is a risk marker for cardiovascular disease, not a risk factor that causes cardiovascular disease. These findings add to previous studies and highlight the need to consider erectile dysfunction in relation to the risk of different types of cardiovascular disease, including heart failure and heart conduction disorders. However, the study's reliance on the answer to a single self-assessed question on erectile functioning limits the findings. Nevertheless, these findings provide useful information for clinicians: men with erectile dysfunction are at higher risk of cardiovascular disease, and the worse the erectile dysfunction, the higher the risk of cardiovascular disease. Men with erectile dysfunction, even at mild or moderate levels, should be screened and treated for cardiovascular disease accordingly.

**Additional Information:**

Please access these websites via the online version of this summary at http://dx.doi.org/10.1371/journal.pmed.1001372.

- Wikipedia defines erectile dysfunction (note that Wikipedia is a free online encyclopedia that anyone can edit)
- MedlinePlus also has some useful patient information on erectile dysfunction
- The Mayo Clinic has patient-friendly information on the causes of, and treatments for, erectile dysfunction, and also includes information on the link with cardiovascular disease
- The National Heart Foundation of Australia provides information for health professionals, patients, and the general public about how to prevent and manage cardiovascular disease, including assessment and management of cardiovascular disease risk

## Introduction

Erectile dysfunction is a common health problem that increases rapidly in prevalence with age. While erectile dysfunction can be a distressing symptom in itself, there is increasing recognition of its importance as a risk marker for potentially life-threatening cardiovascular disease (CVD) events and premature death [1],[2].

CVD is the leading cause of morbidity and mortality globally [3]. Many effective interventions for the primary and secondary prevention of CVD rely on identifying individuals at increased risk. Despite the robustness of current risk prediction models, it is well recognised that a significant proportion of cases of CVD will occur in individuals without classic risk factors [4]. In men without known CVD, erectile dysfunction has potential as a sentinel symptom indicating the presence or increased risk of systemic CVD [1]. Although the possibility has been less widely investigated, erectile dysfunction could also serve as a marker of increased risk of repeated events in men known to have existing CVD.

Currently, evidence on the nature of the relationship of erectile dysfunction to the risk of developing specific subtypes of CVD is limited. Previous prospective studies indicate that, among men without known CVD, those with erectile dysfunction have a significantly increased risk of grouped CVD outcomes, such as coronary heart disease, stroke, and peripheral vascular disease, and all-cause mortality, compared to men without erectile dysfunction [1],[5]–[8]. However, such evidence is limited by the following: the predominant use of dichotomised data on erectile dysfunction status, which precludes evaluation of a dose–response relationship [1],[7]–[9]; the lack of large-scale evidence on how erectile dysfunction relates to different subtypes of CVD; the lack of evidence on how erectile dysfunction relates to CVD in men with existing CVD, who constitute an important high risk group; and the relatively small size and selective nature of most of the studies to date [1],[7],[8],[10].

The aim of this large prospective population-based study is to investigate the relationship of severity of erectile dysfunction, as a marker of risk, to a range of CVD outcomes and all-cause mortality, among men with and without CVD at baseline.

## Methods

### Data Collection and Definitions

The 45 and Up Study is a large-scale Australian cohort study of 123,775 men and 143,378 women aged 45 y and over, randomly sampled from the general population of New South Wales (NSW), Australia. Its methods are described elsewhere [11]. Only men are included in the study presented here. In brief, from 1 February 2006 to 30 April 2009, individuals from the general population of NSW joined the study by completing a postal questionnaire and giving informed consent for follow-up through repeated contact and population health databases.

Data from study participants have been linked probabilistically to the NSW Admitted Patient Data Collection, which is a complete census of all public and private hospital admissions in NSW. It contains details of admissions in participants from 1 July 2000 to 30 June 2010, including dates of admission and discharge, the primary reason for admission using the World Health Organization International Classification of Diseases, 10th revision—Australian Modification (ICD-10-AM) [12], up to 55 additional clinical diagnoses, and up to 50 operations or procedures, coded using Australian Classification of Health Interventions procedure codes [13]. The validity of administrative coding for specific CVD outcomes varies, ranging from fair to very good, with kappa scores for agreement between chart review and recorded diagnoses of 0.6–0.8 [14] and positive predictive values of 66%–99% [15]–[18] for acute myocardial infarction, cerebral infarction, and heart failure. Where misclassification is present, it is unlikely to be biased by prior knowledge of erectile dysfunction status and would therefore tend to result in more conservative estimates of relative risk (RR). Vital status and date of death were ascertained from the date of recruitment up to 31 December 2010 using linkage to the NSW Registry of Births, Deaths and Marriages. Death registrations capture all deaths in NSW. Cause of death information was not available at the time of analysis.

Over the relatively short follow-up period, a small but unknown number of participants are likely to have moved out of the area. Although hospitalisations occurring in neighbouring states would not be captured, these are estimated to make up fewer than 2% of admissions in NSW residents. Hence, follow-up for hospitalisations is considered to be ∼98% complete among those continuing to reside in NSW, and loss to follow-up is considered to be minimal. Quality assurance data show false positive and negative rates for data linkage of <0.5% and <0.1%, respectively.

Baseline questionnaire data included information on socio-demographic factors, lifestyle, height and weight, medical and surgical history, functional capacity, physical activity, and sexual health. The questionnaire for men included a single self-assessment question on erectile functioning: “How often are you able to get and keep an erection that is firm enough for satisfactory sexual activity?”, with the response categories being “always”, “usually”, “sometimes”, “never”, and “I would rather not answer this question”. The first four categories were used to define erectile dysfunction as none, mild, moderate, and severe, respectively. This questionnaire item was based on that used in the Massachusetts Male Aging Study, which has shown good agreement with the International Index of Erectile Function (correlation coefficient = 0.71, kappa = 0.6) [19].

### Statistical Methods

At the time of writing, data from 123,750 male 45 and Up Study participants (99.98%) had been linked to data on hospital admissions and deaths. Among these, men who were missing data (*n* = 4,264; 3.4%), who chose not to answer the question on erectile dysfunction (*n* = 11,034; 8.9%), or whose records had linkage errors (*n* = 25; 0.02%) were excluded. Because cancer and its treatment may lead to erectile dysfunction and affect future survival and CVD risk, those with a self-reported history of prostatectomy and/or cancer other than melanoma and skin cancer (*n* = 13,389; 10.8%) were also excluded. We did not have specific data on other procedures that can result in erectile dysfunction, such as pelvic irradiation, abdomino-pelvic resection, and low anterior resection; however, as these are largely used as treatments for cancer, individuals with these interventions would have been mostly excluded from the analyses. Following exclusions, data were available on 95,038 men for the main analyses.

**Figure 1:**
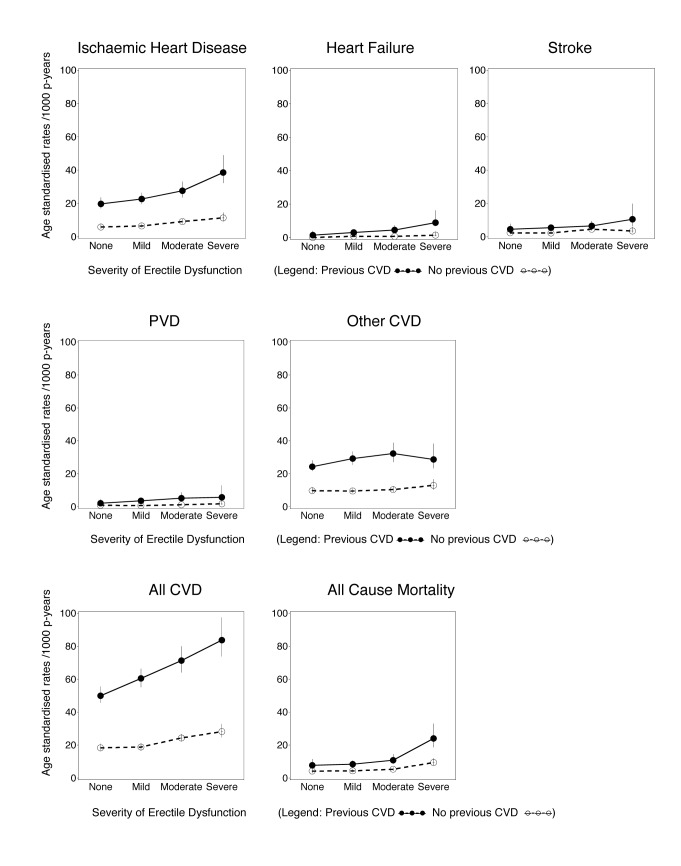
Age-standardised rates (per 1,000 person-years) of grouped CVD hospitalisations since baseline and all-cause mortality by degree of erectile dysfunction and previous CVD history, directly age-standardised to the 2006 New South Wales population. All CVD includes ICD-10-AM diagnosis codes I00–I99, G45, and G46; ischaemic heart disease includes I20–I25; peripheral vascular disease (PVD) includes I70–I74; stroke includes I60–I69, G45, and G46; heart failure is I50; and other CVD is all CVDs except those listed above. Vertical lines indicate 95% CIs.

The main CVD outcome was defined as the first hospitalisation following recruitment into the 45 and Up Study with a primary diagnosis of CVD at discharge, based on ICD-10-AM three-character codes I00–I99, G45, and G46. Diagnoses were grouped consistent with the NSW 2010 health report [20] (see Figure 1), and were also examined individually for diagnoses for which at least 50 events were reported in men with no previous CVD. In the analyses of incident CVD hospitalisation since baseline, eligible men contributed person-years from the date of recruitment until the CVD admission date, date of death, or end of follow-up (30 June 2010), whichever was the earliest. In the analysis of all-cause mortality, eligible men contributed person-years from recruitment until death or end of follow-up (31 December 2010), whichever was the earliest.

Estimates of incident CVD hospitalisation rates since baseline and 95% confidence intervals (95% CIs) were calculated for the different levels of erectile dysfunction at baseline, age-standardised to the 2006 NSW population, in 5-y age groups, using the direct method [21]. Hazard ratios (RRs) for specific CVD outcomes, all CVD, and all-cause mortality according to erectile dysfunction at baseline were estimated using Cox regression modelling, using age as the underlying time variable to finely adjust for age. Each outcome of interest was considered as an end point in the model, except where used for censoring. RRs and 95% CIs take account of age (the underlying time variable), adjusting where appropriate for tobacco smoking (current, past, never), alcohol consumption (0, 1–14, ≥15 drinks/week), marital status (living alone [single/widowed/divorced], living with a partner [married/living with a partner]), annual household pre-tax income (in Australian dollars: <$20,000, $20,000–$39,999, $40,000–$69,999, ≥$70,000), education (secondary school incomplete, secondary school graduation, certificate or diploma, university graduation), tertiles of physical activity (based on number of weekly sessions, weighted for intensity [22]), body mass index (BMI, based on self-reported height and weight, which has been shown to correlate well with that based on measured values in this population [*r* = 0.95], with a relatively small mean difference in values [23]) (15.0–18.49, 18.5–24.99, 25.0–29.99, ≥30.0 kg/m^2^), self-reported doctor-diagnosed diabetes (yes, no), current treatment for hypertension (yes, no), and current treatment for hypercholesterolaemia (yes, no). Missing values for covariates were included in the models as separate categories. No material differences in the main exposure–outcome relationships (ischaemic heart disease and all CVD) were observed when BMI, alcohol consumption, and physical activity were modelled as continuous variables (Table S1). Sensitivity analyses modelling the effect of assuming that men who stated that they would rather not answer the question on erectile dysfunction had severe erectile dysfunction demonstrated that the main findings did not vary materially according to this assumption (Table S2).

Analyses of CVD outcomes were stratified throughout by previous CVD status. Previous CVD was defined as self-reported heart disease, stroke, or blood clot (including peripheral blood clots) on the baseline questionnaire, or a hospital admission in the 6 y prior to study entry with a CVD diagnosis code in any diagnostic field or a CVD-related procedure code in any procedure code field (Table 1).

**Table 1:** Characteristics of study population according to CVD history and degree of erectile dysfunction at baseline.

Characteristic	Degree of Erectile Dysfunction for Individuals with No Previous CVD (*n* = 65,495)				Degree of Erectile Dysfunction for Individuals with Previous CVD (*n* = 29,323)			
	None	Mild	Moderate	Severe	None	Mild	Moderate	Severe
*N*	31,256	17,710	10,561	5,968	7,174	6,492	6,995	8,662
Mean age (years)	55.9 (7.3)	59.5 (8.4)	64.2 (9.5)	71.2 (10.5)	59.8 (8.4)	64.0 (8.9)	68.8 (9.2)	75.1 (9.0)
Ever smoker	44%	48%	55%	58%	52%	56%	60%	63%
≥15 alcoholic drinks/week	25%	27%	26%	23%	24%	25%	23%	19%
Mean BMI (kg/m^2^)	27.0 (3.9)	27.1 (4.1)	27.4 (4.4)	27.1 (4.6)	27.7 (4.2)	27.8 (4.3)	27.8 (4.5)	27.5 (4.8)
Tertiary education	33%	30%	23%	18%	28%	25%	19%	15%
Household income ≥AU$70,000	47%	37%	24%	13%	37%	27%	16%	8%
Married/living with a partner	86%	82%	79%	78%	85%	81%	79%	76%
Highest physical activity tertile	41%	39%	34%	30%	39%	37%	32%	24%
Doctor-diagnosed diabetes	4%	6%	11%	17%	10%	13%	21%	28%
Current treatment for hypertension	13%	18%	25%	29%	29%	35%	39%	41%
Current treatment for hypercholesterolaemia	10%	13%	15%	16%	22%	26%	28%	27%

Data are percent or mean (standard deviation). A past history of CVD at baseline was defined as either self-reported heart disease, stroke, or blood clot on the baseline questionnaire or a hospital admission in the 6 y prior to entering the study with ICD-10-AM diagnosis code I00–I99, G45, or G46 in any of the 55 diagnostic fields or Australian Classification of Health Intervention CVD-related procedure codes (coronary artery bypass angioplasty/stent: 35310, 38306, 35304-00, 30305-00, 38300-00, 38303-00; coronary artery bypass graft: 38497, 38500, 38503, 90201; coronary revascularisation procedures: 38497, 38500, 38503, 90201, 35310, 38306, 35304-00, 30305-00, 38300-00, 38303-00) in any of the 50 procedure code fields.

The proportionality assumption was verified by plotting the Schoenfeld residuals against the time variable in each model, with a stratified form used where covariates displayed non-proportionality of hazards. Tests for trend were performed by modelling erectile dysfunction as an ordinal variable. To test for interaction between erectile dysfunction categories and previous CVD, the likelihood-ratio test was used, stratified by age because of strong age–previous CVD relationships, to compare the model with and without the interaction term. The weighted least-squares method was used to test for heterogeneity in the RR for dichotomised erectile dysfunction (severe/moderate versus mild/none) in different study subgroups. All analyses were carried out using SAS version 9.3 [24]. All statistical tests were two-sided, using a significance level of 5%.

Ethical approval for the study was provided by the NSW Population and Health Services Research Ethics Committee (2010/05/234).

## Results

The study population ranged in age from 45 to 106 y, with a mean of 62.0 y (standard deviation 10.5 y). Erectile dysfunction increased steeply with age, with a prevalence of severe erectile dysfunction of 2.2% at age 45–54 y, 6.8% at age 55–64 y, 20.2% at age 65–74 y, 50.0% at age 75–84 y, and 75.4% at age ≥85 y. Overall, the prevalence of severe erectile dysfunction was higher in smokers and in men with lower incomes, lower consumption of alcohol, lower education, and/or single/widower marital status, compared to other men (Table 1). Severe erectile dysfunction was also more common in men who had been diagnosed with diabetes and/or CVD in the past and in those who were receiving treatment for hypertension and/or hypercholesterolaemia, compared to men without these risk factors.

Median (mean) follow-up time for the cohort was 1.9 (2.2) y for CVD hospitalisation and 2.2 (2.8) y for mortality; over this time, 7,855 incident hospital admissions with a primary diagnosis of CVD and 2,304 deaths occurred. The age-standardised incidences of all-cause mortality and of hospitalisation for ischaemic heart disease, heart failure, peripheral vascular disease, “other” CVD, and all CVD combined generally increased with increasing severity of erectile dysfunction and generally were higher for men with prior CVD at baseline, compared to men without prior CVD (Figure 1).

**Figure 2:**
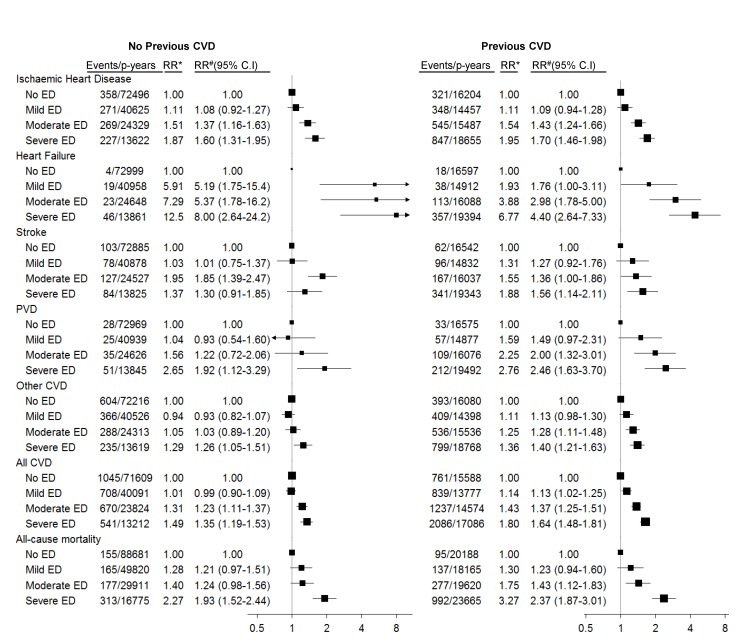
Relative risk of grouped CVD admissions and all-cause mortality since baseline, according to severity of erectile dysfunction at baseline. *RR adjusted for age only. ^#^RR adjusted for age, tobacco smoking, alcohol consumption, marital status, income, education, physical activity, BMI, diabetes, and current treatment for hypertension and hypercholesterolaemia. RRs are plotted on a log scale and are represented with squares with areas inversely proportional to the variance of the logarithm of the RR, providing an indication of the amount of statistical information available; 95% CIs are indicated by horizontal lines. ED, erectile dysfunction, PVD, peripheral vascular disease.

The adjusted RRs of ischaemic heart disease, heart failure, stroke, peripheral vascular disease, “other” CVD, and all CVD combined increased significantly with increasing severity of baseline erectile dysfunction, in men with and without a past history of CVD (Figure 2; *p*[trend]<0.001 for all comparisons, except stroke and other CVD in men without previous CVD, where *p*[trend] = 0.008 and 0.04, respectively). These RRs varied according to the type of CVD outcome examined, with greater RRs for heart failure than for other grouped CVD outcomes (Figure 2). The relationship of severity of erectile dysfunction to CVD outcomes did not vary significantly according to history of CVD prior to baseline (*p*[interaction] for all strata >0.05, except for stroke, where *p*[interaction] = 0.02). Men with severe erectile dysfunction had approximately a doubling in the risk of death during follow-up compared to men without erectile dysfunction (Figure 2), regardless of past CVD history. The relation of erectile dysfunction to subsequent CVD events did not vary materially according to duration of follow-up (i.e., <2 y versus ≥2 y; Table S3).

Among men with no previous history of CVD, significant trends of increasing hospitalisation risk for specific ICD-10-AM primary diagnostic codes with increasing erectile dysfunction were seen for angina pectoris, acute myocardial infarction, chronic ischaemic heart disease, atrioventricular and left bundle branch block, heart failure, and (peripheral) atherosclerosis (Table 2). No clear patterns of increasing risk were observed for conditions such as pulmonary embolism, dysrhythmias, intracerebral haemorrhage, aortic aneurysm, phlebitis and thrombophlebitis, and haemorrhoids. Findings were broadly similar for men with previous CVD (Table 3). There were fewer than 50 admissions for cardiomyopathy (I42) among men without previous CVD, so the findings are not included in Table 2; however, there were more than 50 events for men with previous CVD, with noteworthy findings: RRs (95% CIs) for this outcome were 2.38 (0.95–5.97), 3.61 (1.47–8.84), and 3.21 (1.23–8.35) for mild, moderate, and severe erectile dysfunction versus no dysfunction (*p*[trend] = 0.02).

**Table 2:** Relative risk (95% CI) of incident CVD-related hospital admission since baseline among men with no previous history of CVD by ICD-10-AM diagnosis code, according to degree of erectile dysfunction (ED).

Outcome (ICD-10-AM Code)	Number of Events by ED: None/Mild/Mod/Severe	Adjusted RR (95% CI) for Outcome, according to ED Severity				*p-*Value Trend^a^
		None	Mild	Moderate	Severe	
Transient cerebral ischaemic attacks and related syndromes (G45)	50/27/46/25	1.00	0.78 (0.49–1.26)	1.67 (1.06–2.61)	1.04 (0.58–1.87)	0.3
Essential (primary) hypertension (I10)	11/6/9/4	1.00	0.72 (0.26–1.98)	1.17 (0.44–3.07)	0.61 (0.16–2.35)	0.7
Angina pectoris (I20)	160/105/108/86	1.00	0.94 (0.73–1.21)	1.25 (0.96–1.62)	1.48 (1.08–2.02)	0.009
Acute myocardial infarction (I21)	143/111/104/108	1.00	1.11 (0.86–1.43)	1.30 (0.99–1.70)	1.66 (1.22–2.26)	0.001
Chronic ischaemic heart disease (I25)	120/107/98/83	1.00	1.25 (0.96–1.63)	1.46 (1.09–1.95)	1.74 (1.25–2.42)	<0.001
Pulmonary embolism (I26)	35/20/12/21	1.00	0.85 (0.48–1.49)	0.65 (0.32–1.31)	1.61 (0.83–3.14)	0.5
Other diseases of pericardium (I31)	14/9/3/5	1.00	1.17 (0.49–2.80)	0.52 (0.13–2.04)	0.93 (0.24–3.69)	0.7
Nonrheumatic aortic valve disorders (I35)	9/9/16/15	1.00	1.12 (0.44–2.85)	1.97 (0.82–4.76)	1.91 (0.72–5.02)	0.08
Atrioventricular and left bundle branch block (I44)	4/3/15/16	1.00	0.94 (0.20–4.28)	5.32 (1.60–17.67)	6.62 (1.86–23.56)	<0.001
Paroxysmal tachycardia (I47)	24/28/16/10	1.00	1.79 (1.02–3.12)	1.47 (0.75–2.88)	1.54 (0.66–3.61)	0.2
Atrial fibrillation and flutter (I48)	100/70/77/59	1.00	0.93 (0.68–1.27)	1.22 (0.88–1.68)	1.21 (0.82–1.78)	0.2
Other cardiac arrhythmias (I49)	28/10/11/18	1.00	0.44 (0.21–0.91)	0.53 (0.25–1.15)	0.88 (0.41–1.90)	0.6
Heart failure (I50)	4/19/23/46	1.00	5.19 (1.75–15.45)	5.37 (1.78–16.16)	8.00 (2.64–24.23)	<0.001
Intracerebral haemorrhage (I61)	7/4/14/5	1.00	0.64 (0.18–2.24)	2.08 (0.75–5.79)	0.78 (0.20–2.97)	0.7
Cerebral infarction (I63)	23/25/40/27	1.00	1.30 (0.73–2.32)	2.08 (1.19–3.64)	1.31 (0.67–2.54)	0.2
Stroke, not specified as haemorrhage or infarction (I64)	14/11/17/11	1.00	0.91 (0.40–2.03)	1.39 (0.64–3.05)	0.94 (0.36–2.40)	0.7
Atherosclerosis (I70)^b^	14/13/19/30	1.00	0.96 (0.44–2.06)	1.39 (0.67–2.89)	2.47 (1.18–5.15)	0.009
Aortic aneurysm and dissection (I71)	10/9/13/19	1.00	0.79 (0.32–1.97)	0.96 (0.40–2.30)	1.42 (0.59–3.43)	0.3
Phlebitis and thrombophlebitis (I80)	28/12/15/11	1.00	0.65 (0.33–1.29)	1.13 (0.57–2.23)	1.30 (0.57–2.97)	0.5
Varicose veins of lower extremities (I83)	52/21/17/14	1.00	0.68 (0.40–1.13)	0.87 (0.49–1.57)	1.36 (0.68–2.74)	0.8
Haemorrhoids (I84)	256/141/74/32	1.00	0.99 (0.80–1.22)	0.91 (0.69–1.20)	0.84 (0.56–1.27)	0.4
Hypotension (I95)	6/10/13/12	1.00	1.72 (0.61–4.85)	2.06 (0.72–5.86)	2.03 (0.66–6.28)	0.2

RR adjusted for age, tobacco smoking, alcohol consumption, marital status, income, education, physical activity, BMI, diabetes, and current treatment for hypertension and hypercholesterolaemia. ICD-10-AM three-character codes with at least 50 events among men with no previous history of CVD are reported.

CVD, cardiovascular disease; ED, erectile dysfunction; ICD-10-AM, World Health Organization International Classification of Diseases 10th revision – Australian Modification; RR, relative risk; 95% CI, 95% confidence interval.

a *p*-Values for trend in risk of CVD outcome across the different categories of ED.

b Refers to predominantly peripheral atherosclerosis.

**Table 3:** Relative risk of incident CVD-related hospital admission since baseline among men with previous history of CVD by ICD-10-AM diagnosis code, according to degree of erectile dysfunction.

Outcome (ICD-10-AM Code)	Number of Events by ED: None/Mild/Mod/Severe	Adjusted RR (95% CI) for Outcome, according to ED Severity				*p*-Value Trend^a^
		None	Mild	Moderate	Severe	
Transient cerebral ischaemic attacks and related syndromes (G45)	28/37/51/116	1.00	1.06 (0.64–1.75)	0.88 (0.54–1.44)	0.98 (0.60–1.59)	1.0
Essential (primary) hypertension (I10)	5/9/9/17	1.00	1.57 (0.52–4.75)	1.29 (0.41–4.05)	1.61 (0.51–5.09)	0.9
Angina pectoris (I20)	165/205/289/442	1.00	1.24 (1.00–1.52)	1.44 (1.17–1.77)	1.71 (1.39–2.10)	<0.001
Acute myocardial infarction (I21)	95/74/119/261	1.00	0.75 (0.55–1.03)	0.88 (0.66–1.17)	1.20 (0.90–1.59)	0.05
Chronic ischaemic heart disease (I25)	130/148/234/317	1.00	1.16 (0.92–1.48)	1.62 (1.29–2.04)	1.81 (1.43–2.30)	<0.001
Pulmonary embolism (I26)	22/20/25/36	1.00	0.82 (0.44–1.53)	0.69 (0.37–1.29)	0.59 (0.31–1.12)	0.07
Other diseases of pericardium (I31)	3/5/7/8	1.00	1.71 (0.40–7.25)	1.93 (0.47–8.00)	1.56 (0.34–7.18)	0.9
Nonrheumatic aortic valve disorders (I35)	10/27/33/49	1.00	2.68 (1.28–5.61)	2.40 (1.12–5.12)	2.18 (1.00–4.73)	0.2
Atrioventricular and left bundle branch block (I44)	8/8/23/70	1.00	0.73 (0.27–1.98)	1.25 (0.53–2.93)	2.00 (0.88–4.55)	0.01
Paroxysmal tachycardia (I47)	20/33/27/50	1.00	1.73 (0.98–3.05)	1.10 (0.59–2.04)	1.48 (0.80–2.73)	0.4
Atrial fibrillation and flutter (I48)	143/127/155/262	1.00	0.91 (0.72–1.17)	0.95 (0.74–1.21)	1.18 (0.92–1.52)	0.2
Other cardiac arrhythmias (I49)	13/22/46/62	1.00	1.60 (0.79–3.21)	2.46 (1.27–4.77)	2.03 (1.02–4.03)	0.1
Heart failure (I50)	18/38/113/357	1.00	1.76 (1.00–3.11)	2.98 (1.78–5.00)	4.40 (2.64–7.33)	<0.001
Intracerebral haemorrhage (I61)	3/10/16/27	1.00	2.82 (0.77–10.32)	3.28 (0.92–11.64)	3.35 (0.93–12.09)	0.1
Cerebral infarction (I63)	22/17/59/106	1.00	0.62 (0.33–1.18)	1.39 (0.83–2.34)	1.45 (0.86–2.44)	0.02
Stroke, not specified as haemorrhage or infarction (I64)	8/13/27/67	1.00	1.39 (0.57–3.41)	1.54 (0.67–3.54)	1.95 (0.86–4.42)	0.1
Atherosclerosis (I70)^b^	13/30/65/138	1.00	2.00 (1.04–3.86)	2.97 (1.61–5.50)	3.98 (2.16–7.34)	<0.001
Aortic aneurysm and dissection (I71)	13/19/32/58	1.00	1.20 (0.59–2.45)	1.30 (0.66–2.56)	1.44 (0.73–2.83)	0.3
Phlebitis and thrombophlebitis (I80)	11/5/20/34	1.00	0.47 (0.16–1.36)	1.42 (0.63–3.19)	1.62 (0.70–3.74)	0.1
Varicose veins of lower extremities (I83)	10/25/24/24	1.00	2.71 (1.28–5.72)	2.56 (1.17–5.59)	2.41 (1.04–5.58)	0.1
Haemorrhoids (I84)	132/106/100/65	1.00	1.07 (0.82–1.39)	1.19 (0.90–1.58)	0.83 (0.58–1.19)	0.7
Hypotension (I95)	6/12/37/81	1.00	1.47 (0.55–3.93)	2.51 (1.03–6.07)	2.75 (1.14–6.62)	0.01

RR adjusted for age, tobacco smoking, alcohol consumption, marital status, income, education, physical activity, BMI, diabetes, and current treatment for hypertension and hypercholesterolaemia. ICD-10-AM three-character codes with at least 50 events in men with no previous history of CVD are reported.

a *p*-Values for trend in risk of CVD outcome across the different categories of ED.

b Refers to predominantly peripheral atherosclerosis.

ED, erectile dysfunction.

**Figure 3:**
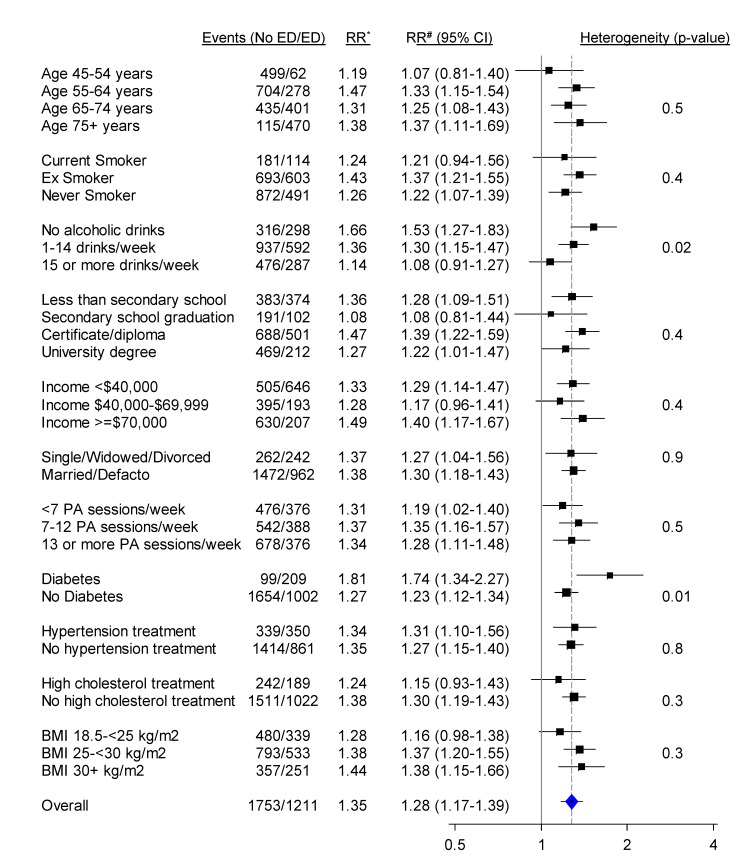
Relative risk of any CVD admission since baseline among men with severe/moderate erectile dysfunction (ED) compared to those with mild/no erectile dysfunction (no ED), in a range of population subgroups, among men with no previous CVD. *RR adjusted for age. #RR adjusted for age, tobacco smoking, alcohol consumption, marital status, income, education, physical activity (PA), BMI, diabetes, and current treatment for hypertension and hypercholesterolaemia where appropriate. RRs are represented with squares with areas inversely proportional to the variance of the logarithm of the RR, providing an indication of the amount of statistical information available. 95% CIs are indicated by horizontal lines. The vertical dotted line represents the overall RR of CVD events in men with moderate/severe erectile dysfunction versus no/mild erectile dysfunction. Defacto, living with a partner.

**Figure 4:**
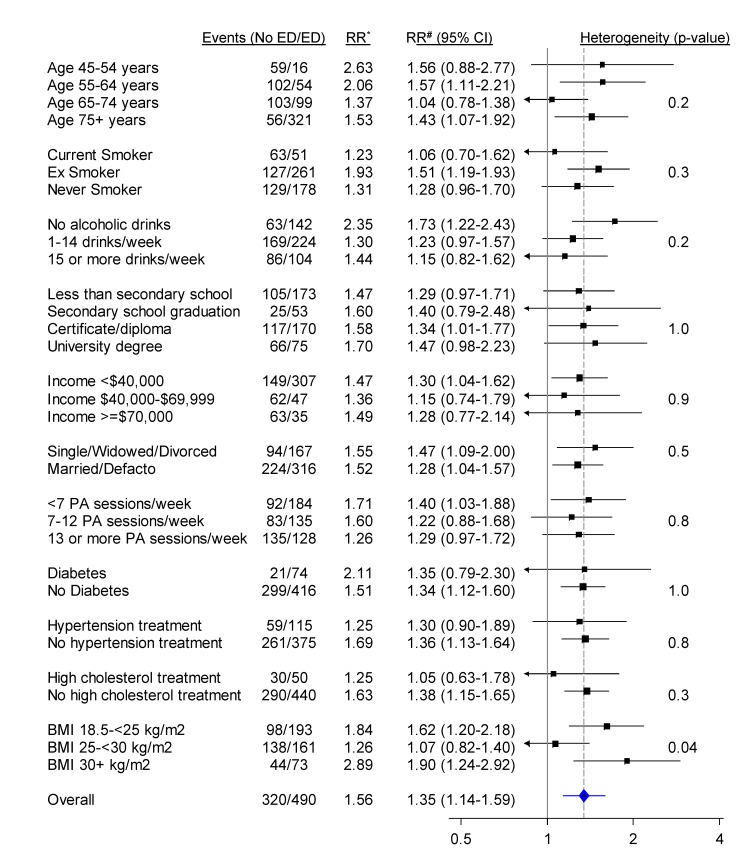
Relative risk of all-cause mortality among men with severe/moderate erectile dysfunction (ED) compared to those with mild/no erectile dysfunction (no ED), in a range of population subgroups, among men with no previous CVD. *RR adjusted for age. #RR adjusted for age, tobacco smoking, alcohol consumption, marital status, income, education, physical activity (PA), BMI, diabetes, and current treatment for hypertension and hypercholesterolaemia where appropriate. RRs are represented with squares with areas inversely proportional to the variance of the logarithm of the RR, providing an indication of the amount of statistical information available. 95% CIs are indicated by horizontal lines. The vertical dotted line represents the overall RR of all-cause mortality in men with moderate/severe erectile dysfunction versus no/mild erectile dysfunction. Defacto, living with a partner.

When categorised as a dichotomous variable (severe/moderate versus no/mild erectile dysfunction), the relationship of erectile dysfunction to all CVD combined was significantly attenuated in those reporting greater alcohol consumption and in those without diabetes, but did not vary significantly (i.e., *p* [heterogeneity] was not significant) by age, smoking, education, income, treatment for hypertension, and a range of other personal characteristics (Figure 3). When adjusted only for age, the relationship of erectile dysfunction to all-cause mortality appeared stronger in younger men (Figure 4). However, this difference was not apparent following more comprehensive adjustment for potential confounding factors (*p*[heterogeneity by age] = 0.2; Figure 4). The association between erectile dysfunction and all-cause mortality did not vary significantly according to any of the factors examined, apart from BMI, where the relationship showed non-systematic variation in the RRs, of borderline statistical significance (*p*[heterogeneity by BMI] = 0.04; Figure 4).

## Discussion

In this large population-based prospective cohort study, the risk of hospitalisation for CVD increased progressively with the self-reported severity of erectile dysfunction in men without and with known CVD at baseline, and this pattern was present after adjusting for established risk factors for CVD. Compared to men without erectile dysfunction, men with severe erectile dysfunction had RRs of approximately 1.5 to 2.0 for ischaemic heart disease, peripheral vascular disease, combined CVD events, and all-cause mortality, and even greater point estimates for risk (albeit with wide confidence intervals) for conditions such as heart failure and atrioventricular and left bundle branch block.

The current study is an order of magnitude larger than any previous prospective study of erectile dysfunction and CVD known to us, and provides the strongest evidence to date of a relationship of increasing CVD risk with increasing self-reported severity of erectile dysfunction [7],[8],[25]. Our results lend strong support to previous studies, which have found that among men without known CVD at baseline, those with moderate or severe erectile dysfunction have increased risks of a subsequent CVD event [1],[7],[8],[10],[25], including ischaemic heart disease [1],[5],[7],[8],[10], stroke [1],[7],[8],[10], and peripheral vascular disease [6],[10], as well as all-cause mortality [7],[26], compared to men with mild or no erectile dysfunction, based on data from over 30,000 participants in relevant prospective studies (∼15,000 in prospective population-based studies) [7],[8]. Although heart failure is often a consequence of ischaemic heart disease, our finding on the relationship between severity of erectile dysfunction and future admissions for heart failure is, to our knowledge, novel and warrants further investigation, given that this condition is common and results in a considerable burden of disease. Similarly, we were unable to locate any previous prospective studies demonstrating the increased risks of chronic ischaemic heart disease and atrioventricular and left bundle branch block with increasing levels of erectile dysfunction.

Erectile dysfunction per se is unlikely to be a major independent cause of CVD, and is best considered as a risk marker rather than a risk factor for CVD. That is, it is likely to serve as an indicator, or “biomarker”, of the severity of underlying pathological processes such as atherosclerosis and endothelial dysfunction. Hence, the study presented here does not aim to investigate erectile dysfunction as an independent cause of CVD. It quantifies the gradient in CVD risk with increasing degrees of erectile dysfunction because this relationship is likely to inform the potential usefulness of erectile dysfunction as a risk marker in predicting events and in discriminating at what level clinical concerns should be raised. Additional CVD risk factors are adjusted for, where possible, with the aim of demonstrating how well the marker predicts risk above and beyond these additional factors.

Although the pathophysiology of erectile dysfunction is multifactorial and includes arterial, neurogenic, hormonal, cavernosal, iatrogenic, and psychogenic causes [27], it is now widely accepted that erectile dysfunction is predominantly due to underlying vascular causes, particularly atherosclerosis [28]. Endothelial dysfunction is considered a key pathophysiological cause of erectile dysfunction and CVD [28]. The normal function of the corpus cavernosum is highly dependent on intact endothelium-dependent relaxation, which potentially explains why erectile dysfunction may precede other clinical manifestations of systemic atherosclerosis. It has also been hypothesized that progressive occlusive/atherosclerotic disease might manifest itself earlier in smaller blood vessels, such as those in the penis, than in larger blood vessels [29]. The observed relationship of erectile dysfunction to subsequent hospitalisation for atherosclerotic conditions such as angina [1], acute myocardial infarction [1], ischaemic stroke, and peripheral vascular disease, over and above known CVD risk factors, is therefore biologically plausible. A large proportion of deaths in older men are attributable to CVD, so the relationship of erectile dysfunction to CVD is likely to be an important contributor to the relationship of erectile dysfunction to all-cause mortality.

The mechanisms underlying the relationship of erectile dysfunction to hospitalisation for the other CVD diagnoses observed in this study require further exploration. Erectile dysfunction is extremely common in men with heart failure [30], and since a large proportion of heart failure is undiagnosed, particularly at its early stages [31], erectile dysfunction may serve as an early marker of occult heart failure that subsequently manifests itself and necessitates treatment in hospital. Conditions categorised as atrioventricular and left bundle branch block are likely to be multifactorial and include ischaemic heart disease as well as fibrotic degeneration of the cardiac conducting system related to primary causes or secondary to ischaemic heart disease and/or cardiomyopathies. The large sample size permitted a large number of comparisons to be performed, which raises type 1 error as a possible explanation for some relationships identified, but the lack of a clear relationship between erectile dysfunction and certain other CVD diagnoses (e.g., pulmonary embolism) suggests a degree of specificity in its potential to predict disease.

The relationship of severity of erectile dysfunction to the different types of CVD was similar for those with and without a prior history of CVD, indicating that erectile dysfunction remains a risk marker even in those with known CVD. Men with existing CVD should, theoretically, already be receiving maximal secondary preventative therapy, so the clinical utility of markers of increased risk of recurrent events is open to question. Since in reality there are consistent and often large gaps between recommended treatment and actual practice for those with existing CVD [32], markers of increased risk of recurrent events could potentially be useful for improving secondary prevention of CVD. However, the findings reported here for individuals with a prior history of CVD should be interpreted with caution, since a number of treatments for CVD increase the risk of erectile dysfunction.

Using data from the cohort prior to exclusions, 16% of men aged 50–59 y, 34% of men aged 60–69 y, and 60% of men aged ≥70 y reported moderate or severe erectile dysfunction; this compares with 11%, 31%, and 68% affected in the same age groups in a representative population-based telephone survey of Australian men [33]. Hence, although representativeness is not necessary for valid and reliable estimates of RR from within-cohort comparisons [34], the degree of erectile dysfunction within the cohort is broadly similar to that in the general population.

Our study included men aged 45 y and over and, following adjustment for a wide range of potential confounding factors, found that the relationship of erectile dysfunction to CVD and to all-cause mortality did not differ significantly for men of different ages. Two previous studies found stronger erectile dysfunction–CVD relationships in younger than in older men [5],[35], particularly in men under 40 y [35], while another found consistent relationships across age groups [36]. In our study, the increased risk of CVD hospitalisation in men with moderate/severe versus mild/no erectile dysfunction was more pronounced in men with diabetes. Other evidence on this is heterogeneous, with previous studies in men with diabetes suggesting slightly lesser and greater erectile-dysfunction-associated risk of CVD compared to studies including the broader population [7],[10]. The attenuation in the erectile dysfunction–CVD relationship with increasing alcohol consumption could potentially be explained by the fact that heavy drinkers may have erectile dysfunction for reasons other than underlying CVD.

In studies of the relationship between risk factors and disease, short follow-up time is often seen as a limitation, particularly if there are issues with reverse causality, where the presence of disease can influence the risk factor itself. In contrast, where a risk marker is being considered, with the aim of measuring something that relates to the presence and severity of underlying disease, short follow-up time is often desirable, as is the case here. Provided there are sufficient numbers of health events, a short follow-up time allows quantification of the relationship of the risk marker to the immediate risk of disease. In particular, because erectile dysfunction status is likely to change increasingly with time since baseline, longer follow-up might lead to reduced accuracy of prediction.

Several study limitations may potentially influence the interpretation of results in our study. (i) While erectile dysfunction at baseline was measured using a single validated question, the duration of dysfunction was unknown. (ii) Data on potential confounding factors were mostly based on self-report. (iii) Measured data on CVD risk factors such as blood pressure and blood lipids were not available. (iv) Information on use of medications, including CVD medications and those used to treat erectile dysfunction, such as phosphodiesterase type 5 inhibitors, was not available for this study. Use of phosphodiesterase type 5 inhibitors is likely to be relatively uncommon in Australia, with the most relevant survey showing that 5% of men aged 40 y and over reported ever having received medical treatment for erectile dysfunction [33]. Given that users may have been misclassified as having no erectile dysfunction, and that use of phosphodiesterase type 5 inhibitors does not appear to influence the risk of myocardial infarction or cardiovascular death [37], this bias, if it were present, would tend to lead to conservative estimates of RR. (v) Administrative data were used for CVD hospitalisation end points—these are independent and virtually complete for cohort members, with fair to very good validity, but were not confirmed individually by chart review or other methods. (vi) Power was limited for some subtypes of CVD. (vii) Finally, we cannot exclude the possibility that the findings presented here are affected by unmeasured confounding. The fact that a range of disease processes, apart from those related to erectile dysfunction, may lead to a CVD event should also be considered.

The findings of this study highlight the need to consider erectile dysfunction in relation to the risk of a wide range of CVDs that extends beyond ischaemic heart disease and stroke and includes conditions such as heart failure and conduction disorders. They also provide evidence that CVD risk is elevated across a spectrum of severity of erectile dysfunction and that men with mild or moderate erectile dysfunction should be considered at increased risk, in addition to those with severe disease. Nevertheless, this does not translate automatically into utility as part of a clinical risk score, such as using erectile dysfunction in addition to the Framingham score [36]. Rather, the findings provide general support for the Princeton consensus [29] that men with erectile dysfunction require assessment for CVD risk, while the quantitative ability of erectile dysfunction to predict risk in the clinical setting, over and above clinically measured risk factors, requires specific testing.

## Supporting Information

Table S1 Sensitivity analysis: adjusted relative risk of ischaemic heart disease admissions and all CVD admissions, according to erectile dysfunction severity at baseline, in men without previous CVD, with specification of certain variables as continuous or categorical.
(DOC)

Table S2 Sensitivity analysis: adjusted relative risk of various CVD events according to degree of erectile dysfunction severity at baseline, in men without previous CVD, with and without imputation of data for men recording “do not wish to answer” for the question on erectile dysfunction.
(DOC)

Table S3 Sensitivity analysis: adjusted relative risk of ischaemic heart disease admissions and all CVD admissions, according to severity of erectile dysfunction at baseline, in men without previous CVD, for differing durations of follow up.
(DOCX)

## Abbreviations

BMI: body mass index
95% CI: 95% confidence interval
CVD: cardiovascular disease
ICD-10-AM: International Classification of Diseases, 10th revision—Australian Modification
NSW: New South Wales
RR: relative risk
