# Supplementary material for: Erectile Dysfunction Severity as a Risk Marker for Cardiovascular Disease Hospitalisation and All-Cause Mortality: A Prospective Cohort Study
Source: PLoS Med. 2013 Jan 29;10(1):e1001372. doi: 10.1371/journal.pmed.1001372 (PMC3558249; doi:10.1371/journal.pmed.1001372)
Supplement: Table S1 — Sensitivity analysis: adjusted relative risk of ischaemic heart disease admissions and all CVD admissions, according to erectile dysfunction severity at baseline, in men without previous CVD, with specification of certain variables as continuous or categorical. (DOC) [file pmed.1001372.s001.doc]

**Table S1. Sensitivity analysis: adjusted relative risk of ischaemic heart disease admissions and all CVD admissions, according to erectile dysfunction severity at baseline, in men without previous CVD, with specification of certain variables as continuous or categorical.**

|  |  | **Adjusted relative risk* (95%CI) of specified outcome** | | | |
| --- | --- | --- | --- | --- | --- |
|  |  | All covariates categorical** | BMI continuous  All other covariates categorical | BMI, ADPW continuous  All other covariates categorical | BMI, ADPW, PAS continuous  All other covariates categorical |
| **Ischaemic Heart Disease (events)** | | (1125) | (1048) | (1030) | (995) |
|  | No erectile dysfunction | 1.00 | 1.00 | 1.00 | 1.00 |
|  | Mild erectile dysfunction | 1.08 (0.92-1.27) | 1.05 (0.89-1.24) | 1.05 (0.89-1.25) | 1.04 (0.87-1.23) |
|  | Moderate erectile dysfunction | 1.37 (1.16-1.63) | 1.35 (1.13-1.61) | 1.37 (1.15-1.64) | 1.36 (1.13-1.63) |
|  | Severe erectile dysfunction | 1.60 (1.31-1.95) | 1.62 (1.32-1.99) | 1.67 (1.36-2.06) | 1.71 (1.38-2.11) |
|  |  |  |  |  |  |
| **All CVD (events)** | | (2964) | (2773) | (2727) | (2617) |
|  | No erectile dysfunction | 1.00 | 1.00 | 1.00 | 1.00 |
|  | Mild erectile dysfunction | 0.99 (0.90-1.09) | 0.96 (0.87-1.07) | 0.96 (0.87-1.07) | 0.96 (0.87-1.07) |
|  | Moderate erectile dysfunction | 1.23 (1.11-1.37) | 1.23 (1.10-1.37) | 1.24 (1.11-1.39) | 1.24 (1.11-1.39) |
|  | Severe erectile dysfunction | 1.35 (1.19-1.53) | 1.37 (1.21-1.57) | 1.38 (1.21-1.58) | 1.41 (1.23-1.61) |

CVD=cardiovascular disease, BMI=body mass index, ADPW=alcoholic drinks per week, PAS= weighted physical activity sessions per week,

*Relative risk adjusted for age, tobacco smoking, alcohol consumption, marital status, income, education, physical activity, BMI, diabetes and current treatment for hypertension and hypercholesterolaemia.

** As shown in Figure 2, with age modelled as the underlying time variable and all other covariates modelled as categorical, including missing values modelled as a separate category
