# Supplementary material for: Erectile Dysfunction Severity as a Risk Marker for Cardiovascular Disease Hospitalisation and All-Cause Mortality: A Prospective Cohort Study
Source: PLoS Med. 2013 Jan 29;10(1):e1001372. doi: 10.1371/journal.pmed.1001372 (PMC3558249; doi:10.1371/journal.pmed.1001372)
Supplement: Table S2 — Sensitivity analysis: adjusted relative risk of various CVD events according to degree of erectile dysfunction severity at baseline, in men without previous CVD, with and without imputation of data for men recording “do not wish to answer” for the question on erectile dysfunction. (DOC) [file pmed.1001372.s002.doc]

**Table S2. Sensitivity analysis: adjusted relative risk of various CVD events according to degree of erectile dysfunction severity at baseline, in men without previous CVD, with and without imputation of data for men recording “do not wish to answer” for the question on erectile dysfunction.**

|  |  | **Adjusted relative risk* (95%CI) of specified outcome** | |
| --- | --- | --- | --- |
|  |  | Original results**  n=65,495 | Imputation of “do not wish to answer”  as severe erectile dysfunction  n=72,524 |
| **Ischaemic Heart Disease** | | | |
|  | No erectile dysfunction | 1.00 | 1.00 |
|  | Mild erectile dysfunction | 1.08 (0.92-1.27) | 1.09 (0.93-1.28) |
|  | Moderate erectile dysfunction | 1.37 (1.16-1.63) | 1.38 (1.17-1.64) |
|  | Severe erectile dysfunction | 1.60 (1.31-1.95) | 1.62 (1.37-1.92) |
| **Heart Failure** | | | |
|  | No erectile dysfunction | 1.00 | 1.00 |
|  | Mild erectile dysfunction | 5.19 (1.75-15.45) | 5.48 (1.85-16.22) |
|  | Moderate erectile dysfunction | 5.37 (1.78-16.16) | 5.79 (1.95-17.23) |
|  | Severe erectile dysfunction | 8.00 (2.64-24.23) | 8.96 (3.11-25.78) |
| **Peripheral Vascular Disease** | | | |
|  | No erectile dysfunction | 1.00 | 1.00 |
|  | Mild erectile dysfunction | 0.93 (0.54-1.60) | 0.91 (0.53-1.57) |
|  | Moderate erectile dysfunction | 1.22 (0.72-2.06) | 1.15 (0.68-1.93) |
|  | Severe erectile dysfunction | 1.92 (1.12-3.29) | 1.64 (1.01-2.65) |
| **Stroke** | | | |
|  | No erectile dysfunction | 1.00 | 1.00 |
|  | Mild erectile dysfunction | 1.01 (0.75-1.37) | 1.00 (0.74-1.35) |
|  | Moderate erectile dysfunction | 1.85 (1.39-2.47) | 1.85 (1.39-2.46) |
|  | Severe erectile dysfunction | 1.30 (0.91-1.85) | 1.49 (1.11-2.00) |
| **Other CVD** | | | |
|  | No erectile dysfunction | 1.00 | 1.00 |
|  | Mild erectile dysfunction | 0.93 (0.82-1.07) | 0.93 (0.81-1.06) |
|  | Moderate erectile dysfunction | 1.03 (0.89-1.20) | 1.01 (0.87-1.18) |
|  | Severe erectile dysfunction | 1.26 (1.05-1.51) | 1.27 (1.09-1.46) |
| **All CVD** | | | |
|  | No erectile dysfunction | 1.00 | 1.00 |
|  | Mild erectile dysfunction | 0.99 (0.90-1.09) | 0.99 (0.90-1.09) |
|  | Moderate erectile dysfunction | 1.23 (1.11-1.37) | 1.22 (1.10-1.35) |
|  | Severe erectile dysfunction | 1.35 (1.19-1.53) | 1.39 (1.25-1.54) |
| **All-cause mortality** | | | |
|  | No erectile dysfunction | 1.00 | 1.00 |
|  | Mild erectile dysfunction | 1.21 (0.97-1.51) | 1.18 (0.95-1.48) |
|  | Moderate erectile dysfunction | 1.24 (0.98-1.56) | 1.18 (0.94-1.48) |
|  | Severe erectile dysfunction | 1.93 (1.52-2.44) | 1.75 (1.43-2.16) |

CVD=cardiovascular disease
*Relative risk adjusted for age, tobacco smoking, alcohol consumption, marital status, income, education, physical activity, body mass index, diabetes and current treatment for hypertension and hypercholesterolaemia.

**As shown in Figure 2.
