# Supplementary material for: Erectile Dysfunction Severity as a Risk Marker for Cardiovascular Disease Hospitalisation and All-Cause Mortality: A Prospective Cohort Study
Source: PLoS Med. 2013 Jan 29;10(1):e1001372. doi: 10.1371/journal.pmed.1001372 (PMC3558249; doi:10.1371/journal.pmed.1001372)
Supplement: Table S3 — Sensitivity analysis: adjusted relative risk of ischaemic heart disease admissions and all CVD admissions, according to severity of erectile dysfunction at baseline, in men without previous CVD, for differing durations of follow up. (DOCX) [file pmed.1001372.s003.docx]

**Table S3. Sensitivity analysis: adjusted relative risk of ischaemic heart disease admissions and all CVD admissions, according severity of erectile dysfunction at baseline, in men without previous CVD, for differing durations of follow up.**

|  |  |  | **Events** | **Person-years** | **Adjusted Relative risk* (95%CI)**  **of specified outcome** | | |
| --- | --- | --- | --- | --- | --- | --- | --- |
| **Ischaemic Heart Disease** | | |  |  | |  |  |
|  | *Follow up ≤ 2 years* | |  |  | |  |  |
|  |  | No erectile dysfunction | 289 | 30536 | | 1.00 |  |
|  |  | Mild erectile dysfunction | 219 | 17475 | | 1.05 (0.87-1.25) |  |
|  |  | Moderate erectile dysfunction | 216 | 10193 | | 1.32 (1.09-1.59) |  |
|  |  | Severe erectile dysfunction | 182 | 5634 | | 1.47 (1.18-1.84) |  |
|  | *Follow up > 2 years* | |  |  | |  |  |
|  |  | No erectile dysfunction | 69 | 41960 | | 1.00 |  |
|  |  | Mild erectile dysfunction | 52 | 23151 | | 1.07 (0.74-1.54) |  |
|  |  | Moderate erectile dysfunction | 53 | 14136 | | 1.34 (0.91-1.97) |  |
|  |  | Severe erectile dysfunction | 45 | 7988 | | 1.37 (0.86-2.20) |  |
|  | *Total*** | |  |  | |  |  |
|  |  | No erectile dysfunction | 358 | 72496 | | 1.00 |  |
|  |  | Mild erectile dysfunction | 271 | 40625 | | 1.08 (0.92-1.27) |  |
|  |  | Moderate erectile dysfunction | 269 | 24329 | | 1.37 (1.16-1.63) |  |
|  |  | Severe erectile dysfunction | 227 | 13622 | | 1.60 (1.31-1.95) |  |
|  | | |  |  | |  |  |
| **All CVD** | | |  |  | |  |  |
|  | *Follow up ≤ 2 years* | |  |  | |  |  |
|  |  | No erectile dysfunction | 850 | 30564 | | 1.00 |  |
|  |  | Mild erectile dysfunction | 570 | 17464 | | 0.95 (0.85-1.06) |  |
|  |  | Moderate erectile dysfunction | 537 | 10211 | | 1.16 (1.03-1.30) |  |
|  |  | Severe erectile dysfunction | 448 | 5649 | | 1.28 (1.11-1.48) |  |
|  | *Follow up > 2 years* | |  |  | |  |  |
|  |  | No erectile dysfunction | 195 | 41045 | | 1.00 |  |
|  |  | Mild erectile dysfunction | 138 | 22627 | | 1.01 (0.81-1.27) |  |
|  |  | Moderate erectile dysfunction | 133 | 13613 | | 1.24 (0.98-1.58) |  |
|  |  | Severe erectile dysfunction | 93 | 7564 | | 1.12 (0.83-1.52) |  |
|  | *Total*** | |  |  | |  |  |
|  |  | No erectile dysfunction | 1045 | 71610 | | 1.00 |  |
|  |  | Mild erectile dysfunction | 708 | 40091 | | 0.99 (0.90-1.09) |  |
|  |  | Moderate erectile dysfunction | 670 | 23824 | | 1.23 (1.11-1.37) |  |
|  |  | Severe erectile dysfunction | 541 | 13212 | | 1.35 (1.19-1.53) |  |

CVD=cardiovascular disease,

*Relative risk adjusted for age, tobacco smoking, alcohol consumption, marital status, income, education, physical activity, body mass index, diabetes and current treatment for hypertension and hypercholesterolaemia.

**Original results, as shown in Figure 2.
